# Developing medical artificial intelligence leaders: International university consortium approach

**DOI:** 10.1111/medu.14635

**Published:** 2021-08-31

**Authors:** Young‐Mee Lee, Khoa Cao, Michelle Leech, Fabrizio De Ponti

## 1 WHAT PROBLEM WAS ADDRESSED?

Machine learning applications are increasingly used in medicine. The demand for ‘augmented doctors,’^1^ or ‘enhanced physicians’, that is, physicians capitalising rather than opposing incoming technology, has been recognised. However, teaching medical Artificial Intelligence is challenging, especially in already oversaturated medical curricula with most medical schools lacking Artificial Intelligence expertise.

To help medical students become ‘augmented doctors’, an international collaborative educational project, the GAME‐TEI (Global Alliance of Medical Excellence–Transnational Educational Initiative) (https://www.game-med.net/tei), planned its ‘summer school’ themed *Artificial Intelligence in Medicine and Medical Education* at the University of Bologna, Italy in July 2020. The COVID‐19 pandemic led to a rapid transformation into an online course involving medical students from nine countries.

## 2 WHAT WAS TRIED?

The international online medical Artificial Intelligence summer school ran a 6‐week asynchronous pre‐program followed by 3 days of live workshops. Forty‐five medical students from representative medical schools (Asia‐Pacific 18, Europe 20 and Canada 7) participated. Prior Artificial Intelligence experience was a desirable factor as it was originally intended as a 1‐week intensive course. The online format allowed expanded student numbers and inclusion of Artificial Intelligence novice participants as well as a longer duration and a sequence of elements that better supported learning.

During the online 6‐week asynchronous programme, students chose between *Foundations of Medical Artificial Intelligence* or *Advanced Medical Artificial Intelligence* aligning the coursework with the students' perceived level of competency. Both streams included series of lectures and interactive sessions (total 12 sessions, 2 h/each) covering medical Artificial Intelligence engineering, ethics and policy. The programme was grounded in project‐based learning, with a competitive task to develop an Artificial Intelligence tool and a literature review in the student's field of interest. Students worked in small international teams to complete a team‐based Digital Biodesign process to identify a real‐world need and pitch a feasible medical Artificial Intelligence application to colleagues and faculty panel.

Students' percieved competency in medical Artificial Intelligence was assessed pre‐ and post‐programme, based on predetermined course outcomes (5‐point Likert scales), 86.6% of participants responded to both surveys. Overall satisfaction with the Artificial Intelligence course was high (4.58 ± 0.46) with the scores for lectures (4.80 ± 0.46), assignments (4.48 ± 0.68) and live workshops (4.45 ± 0.85). Students' self‐assessed competency in Artificial Intelligence showed a striking increase in all Artificial Intelligence learning outcomes (*p* < .000).

## 3 WHAT LESSONS WERE LEARNED

The online teaching format maximised student learning experience in Medical Artificial Intelligence and minimised the costs of an international course. A dual approach with an asynchronous program and synchronous workshops, interactive tutoring, peer tutoring and self‐directed learning contributed to the successful implementation of the programme. The online medium allowed us to scale up and involve world class experts. Several limitations of online format were identified. Time differences hindered simultaneous gathering of all 45 students, and the learning needs from different cultures/systems, and human interactions could not be fully satisfied. Artificial Intelligence is of great interest to medical students, and global trends in Artificial Intelligence and medicine demand some attention to this in curricula. In the resource constrained tertiary education sector in many countries, a university consortium approach may well address the building of future medical leaders in Artificial Intelligence and Health.
